# Association between Intention to Quit Cigarette Smoking and Use of Heated Tobacco Products: Application of Smoking Intensity Perspective on Heated Tobacco Product Users

**DOI:** 10.3390/ijerph17228471

**Published:** 2020-11-16

**Authors:** Dong-Hee Ryu, Soon-Woo Park, Jun Hyun Hwang

**Affiliations:** Department of Preventive Medicine, Catholic University of Daegu School of Medicine, Daegu 42472, Korea; ryudh@cu.ac.kr (D.-H.R.); parksw@cu.ac.kr (S.-W.P.)

**Keywords:** intention to quit smoking, heated tobacco products, cigarettes

## Abstract

Tobacco companies have designed sophisticated marketing strategies for heated tobacco products (HTPs), and many smokers are exposed to advertisements purporting that HTPs can replace combustible cigarettes. The present study evaluates the relationship between poly-use of tobacco products and intention to quit cigarette smoking in association with smoking intensity, a meaningful indication of one’s interest in quitting cigarette smoking. A total of 36,397 current cigarette smokers who participated in the 2019 Korea Community Health Survey were evaluated. A multivariable logistic regression model was designed. Additionally, smoking-intensity-stratified analyses were conducted. A total of 4.7% of the participants reported planning to quit cigarette smoking within one month. Current dual users of combustible cigarettes and HTPs presented no significantly increased likelihood of intention to quit cigarette smoking regardless of cigarette-smoking intensity. By contrast, light and heavy daily smokers who accompanied e-cigarette use presented significant adjusted odds ratios (aORs) of 1.81 (95% confidence interval (CI): 1.04–3.14) and 1.97 (95% CI: 1.14–3.42), respectively. Occasional and daily smokers who reported using both HTPs and e-cigarettes presented no significance. The results of the present study suggested that a complete replacement of combustible cigarettes with HTPs was unlikely.

## 1. Introduction

The World Health Organization stated that “global targets for reducing tobacco will not be reached unless current tobacco users quit”, emphasizing that quitting tobacco has major and immediate health benefits [1]. One of the most important factors predicting quitting attempts is intention to quit [2,3,4]. Intention represents a mental determination to perform a certain action; people with intentions to perform an action are more likely to make efforts to achieve their goals [5]. According to PRIME (i.e., plans, responses, impulses, motives, evaluations) theory, intention to quit smoking in the near future (plan) is a motivation that positively influences the adoption of cessation behaviors [3].

Recent enthusiasm for new types of tobacco products has made it more complicated to interpret smokers’ intention to quit cigarette smoking. Of the new types of tobacco products, heated tobacco products (HTPs) and electronic cigarettes (e-cigarettes) are among the most common [6]. HTPs, also known as “heat-not-burn”, are non-combustible devices that generate a nicotine-containing aerosol by heating processed tobacco [7]. By contrast, e-cigarettes are products that heat a nicotine-containing solution mainly comprising propylene glycol or glycerol [8]. The key elements of marketing strategies adopted by the leading companies in the tobacco industry when launching these products can be summarized as “harm reduction” and “replacements for traditional cigarettes”. Although Philip Morris International (PMI) states that one should quit cigarette smoking completely or switch to an alternative to cigarettes [9], switching completely from cigarettes to an alternative seems uncommon. Tabuchi et al. [10] reported that 72% of users of HTPs or e-cigarettes are dual users of traditional cigarettes, and more recently, another study found that the proportions of dual or triple users among people who use HTPs and e-cigarettes are 96.25% and 93.23%, respectively [11]. Moreover, “complete switching” means that one should still be using another type of tobacco product that is not a combustible cigarette. These marketing strategies give consumers a sense that the new types of tobacco products represent a healthier means of smoking and they should not necessarily view using these products as real smoking. The long-term clinical consequences of novel tobacco products cannot be fully understood until ample time passes to conduct comprehensive longitudinal studies. Therefore, it is necessary to examine intention to quit cigarette smoking among current smokers in detail.

Another aspect to consider with regard to cigarette-quitting intent is its association with cigarette-smoking intensity. Emery et al. [12] suggested that smokers’ current addiction levels, quitting history, and intentions to quit in the future are three major indicators of quitting behaviors. Notably, people with higher nicotine dependence are less likely to make quitting attempts [13,14] or to even have any intention to quit cigarette smoking [15]. Therefore, smoking intensity, a meaningful indication of one’s interest in quitting smoking, should be considered when examining use of novel tobacco products in association with facilitating smoking cessation. There have been numerous studies that evaluate the association between e-cigarette use and intention to quit cigarette smoking, some of which included an in-depth analysis in relation to cigarette-smoking frequency (e.g., daily versus non-daily smoking) [16,17] or daily cigarette consumption (e.g., number of cigarettes smoked per day) [18,19]. Meanwhile, other studies focused on the association between HTP use and intention to quit cigarette smoking [20,21,22]. While previous studies of HTPs have considered the association with regard to cigarette-smoking frequency, they have not comprehensively evaluated the influence of cigarette-smoking intensity.

Therefore, the present study evaluates the association between poly-use of tobacco products and intention to quit cigarette smoking, and the association is then further investigated with regard to smoking intensity.

## 2. Materials and Methods

### 2.1. Data Source

In 2008, the Korea Centers for Disease Control and Prevention (KCDC) initiated the Korea Community Health Survey and performed nationwide data collection [23]. The Korean territory comprises 17 administrative divisions, which are further subdivided into 255 entities based on community-health-center locations. Stratified multistage probability sampling was used for sample selection, and approximately 900 subjects were recruited from each community. Well-trained interviewers visited the selected sample houses and conducted one-on-one interviews with all adults aged 19 years or older using the computer-assisted personal interview method. All participants provided written informed consent, and the survey was approved by the Institutional Review Board of the KCDC; detailed information regarding this survey is available elsewhere [23]. The present study used the 2019 data from the Korea Community Health Survey (KCHS), which were collected from August to October 2019. Of the 229,099 participants involved in the 2019 wave, 37,943 individuals reported being current smokers. This study used data for 36,397 individuals who provided no missing data for key variables.

### 2.2. Study Variables

In the present study, “current cigarette smokers” were defined as people who had smoked at least 100 cigarettes during their lifetime and who were currently (i.e., at the time of the survey) smoking either daily or occasionally. Participants were categorized into four groups based on their current use of HTPs and/or e-cigarettes: exclusive cigarette smoker, dual smoker with HTPs, dual smoker with e-cigarettes, and triple smoker. This was performed through the following process. People who were currently (i.e., at the time of the survey) using HTPs either daily or occasionally were defined as “current HTP users”. People who reported using e-cigarettes within the past month were defined as “current e-cigarette users”. Then, current cigarette smokers who had not currently used either HTPs or e-cigarettes were defined as “exclusive cigarette smokers”, while current cigarette smokers who reported current use of both HTPs and e-cigarettes were defined as “triple users”. Current cigarette smokers who had currently used HTPs but not e-cigarettes were defined as “dual smokers with HTPs”, and those who currently used e-cigarettes but not HTPs were defined as “dual smokers with e-cigarettes”.

Additionally, current cigarette smokers were further categorized into occasional, light daily (<10 cigarettes per day), moderate daily (10~19 cigarettes per day), and heavy daily (≥20 cigarettes per day) smokers based on their cigarette-smoking intensity.

The dependent variable was intention to quit cigarette smoking within one month. Study subjects were asked “are you planning to quit smoking?” and given the following four response options: “within the next month”, “within the next six months”, “sometime in the future beyond six months”, and “not planning to quit smoking”. Respondents who reported planning to quit smoking “within the next month” were defined as subjects with intention to quit smoking.

Covariates included individual and health-related characteristics. The individual characteristics comprised sex, age, monthly household income, education, and residential area. The health-related variables were subjective health status, stress level, and monthly alcohol consumption. Study subjects had used specialized response options to self-report their stress levels (“none at all”, “very little”, “somewhat”, and “high”) and health status (“excellent”, “ good”, “fair”, “poor”, and “very poor”). Based on these data, subjective health status was further categorized into three groups: “excellent/good”, “fair”, “poor/very poor”. Meanwhile, monthly alcohol consumption was classified into three categories: “none” (no alcohol consumption within the past month), “light–moderate” (some alcohol consumption within the past month, but less than that of heavy drinkers), and “heavy” (seven or more drinks for males and five or more drinks for females in a single session at least two days per week).

### 2.3. Statistical Analysis

Weighted percentages were calculated to examine intention to quit cigarette smoking in terms of tobacco product use and personal characteristics. A multivariable logistic regression model was designed to evaluate the relationship between tobacco product use and intention to quit cigarette smoking. Current poly-use of tobacco products was included as the main effective variable in the logistic model and was adjusted for all covariates. Additionally, smoking-intensity-stratified analyses were conducted. All statistical analyses were performed using SPSS version 19.0 (BMI, Armonk, NY, USA) and *p*-values of <0.05 were considered to indicate statistical significance.

## 3. Results

Table 1 shows participants’ intention to quit cigarette smoking within one month in terms of tobacco product use and participants’ characteristics. A total of 4.7% of the current cigarette smokers reported that they were planning to quit smoking within one month. In terms of type of smoking, the weighted percentage of intention to quit was lowest among dual users of combustible cigarettes and HTPs (4.3%). An indirectly proportional relationship was detected between cigarette-smoking intensity and intention to quit. In terms of cigarette-smoking intensity, the weighted percentage of intention to quit was highest among occasional smokers, at 16.0%, and it was lowest among heavy daily smokers, at 2.7%.

Table 2 shows the relationship between tobacco product use and intention to quit cigarette smoking within one month; exclusive cigarette smokers were set as a reference. Respondents who were currently using combustible cigarettes and HTPs together presented an adjusted odds ratio (aOR) of 0.80, but this was not statistically significant (95% confidence interval (CI): 0.62–1.04). Dual users of combustible cigarettes and e-cigarettes were significantly more likely to have an intention to quit (aOR: 1.48, 95% CI: 1.13–1.95) when compared to exclusive cigarette smokers. Triple users showed an aOR of 1.15 (95% CI: 0.85–1.54). There were positive associations between intention to quit cigarette smoking and being male, being 19–29 years of age, having junior high-school-level education or higher, and having fair or poor/very poor self-rated health.

Table 3 shows the results of multivariable logistic regression analyses in association with smoking intensity. Current dual users of combustible cigarettes and HTPs presented no significantly increased likelihood of intention to quit cigarette smoking regardless of cigarette-smoking intensity. Light and heavy daily smokers who accompanied e-cigarette use presented significant aORs of 1.81 (95% CI: 1.04–3.14) and 1.97 (95% CI: 1.14–3.42), respectively. Occasional and daily smokers who reported using both HTPs and e-cigarettes presented no significance. Appendix A (Table A1) shows the number of participants and their cigarette quit intents according to tobacco product use and cigarette-smoking intensity.

## 4. Discussion

Korea is a particularly intriguing region for studying new types of tobacco products, as both HTPs and e-cigarettes are relatively common. Since PMI introduced an HTP named IQOS^®^ in Korea in June 2017 [24], IQOS^®^ had rapidly gained popularity in the market. Subsequently, Korea became the world’s number-two heated tobacco market in 2018 [25]. Since the government recommended refraining from the use of e-cigarettes concerning “e-cigarette, or vaporing, product use-associated lung injury” (also known as “EVALI”), the sales rates of e-cigarettes have dropped. The figure in the first quarter of 2020 was the lowest since the launch of e-cigarettes in 2011 [26]. Meanwhile, the popularity of HTPs in the Korean tobacco market has not cooled down.

Tobacco companies have designed sophisticated marketing strategies for HTPs, and many smokers are exposed to advertisements purporting that HTPs can replace combustible cigarettes [27]. However, the results of the present study suggested that a complete replacement of combustible cigarettes with HTPs was unlikely. Compared to exclusive cigarette smokers, current dual users of combustible cigarettes and HTPs showed no significant intention to quit cigarette smoking within one month regardless of their cigarette-smoking intensity. A previous focus group interview conducted in Korea showed that males used HTPs to avoid family members’ pressure to quit or to smoke in non-smoking areas while females used them to avoid social stigma associated with female smoking or to smoke outdoors [28]. Likewise, HTPs may be perceived by many smokers as a means of enjoying tobacco taste with lower risk [29]. Even though 12.3% of current smokers reported poly-use of HTPs, the present study results indicate a necessity to regulate such advertisements. Considering current product popularity, there is a possibility of increase in the number of individuals who choose such products believing a replacement of combustible cigarettes is possible as the advertisements claim. Moreover, approximately 4.0% (weighted percentage) of former smokers reported current use of HTPs (data not shown). This looks like a complete switching, but whether such use of another nicotine product occurred newly after complete smoking cessation is unknown. Further studies are needed to investigate factors associated with brand-new uses of HTPs in former smokers.

In addition, the present study indicated that smokers’ interest in quitting was quite low; a total of 4.7% (weighted percentage; unweighted percentage, 4.3%) of current smokers reported that they were interested in quitting cigarette smoking within one month. In a previous study using the 2014 KCHS data, the unweighted percentage of current smokers who reported intention to quit cigarette smoking within one month was 8.5% [19]. There seems to be a recent decrease of the figure among Koreans. Therefore, further studies evaluating the intersection of HTP use, motivations of product selection or continuous use, and cessation-associated behaviors are required, and appropriate measures to improve prevalence of having intention to quit cigarette smoking among current smokers are also necessary.

On the other hand, dual smokers with combustible cigarettes and e-cigarettes were slightly more likely to intend to quit cigarette smoking. This was inconsistent with a longitudinal research conducted in the US; the study figured that ever-use of e-cigarettes was not associated with successful cessation or reduced cigarette consumption, even among heavy smokers [18]. Other studies based on large-sized population also showed that e-cigarette use was not associated with smoking cessation [30,31,32]. By contrast, the present study result was consistent with a previous study conducted in Korea, which reported that smokers with intention to quit cigarette smoking were more likely to have tried e-cigarettes or to currently use them [19]. In addition, daily smokers accompanying e-cigarette use presented increased likelihood of intention to quit cigarette smoking regardless of the number of cigarettes consumed per day. Notably, there were statistical significances in light daily and heavy daily smokers. Since the 2019 KCHS data did not provide related information, e-cigarette use intensity was not thoroughly considered in the present study, so this might have caused the insignificance detected in moderate daily smokers. A longitudinal study reported that the odds of successful cessation at follow-up were higher among daily dual users of combustible cigarettes and e-cigarettes than occasional dual users [33]. Therefore, the present study was unable to address whether e-cigarettes can truly be used as an indicator for intention to quit cigarette smoking.

The present study has several limitations. First, intention to quit was used as a proxy for actual quitting. Additional studies are required in order to assess the effect of HTPs on successful smoking cessation. Second, since this study used cross-sectional survey data, causal inferences cannot be derived. Third, no details about HTP or e-cigarette use were provided. It would have been helpful if information such as duration, product types, and motivation of use were included in the survey. Fourth, other techniques such as formal cessation counseling programs, interventions by clinicians, or support from laypersons that might influence a smoker’s intention to quit were not considered. Despite these limitations, the present study is the first that examined the association between intention to quit and HTP use with a focus on cigarette-smoking intensity using nationally representative data.

## 5. Conclusions

The results of the present study suggested that a complete replacement of combustible cigarettes with HTPs was unlikely. This implies that advertisements and marketing strategies designed to convince consumers that a complete replacement of combustible cigarettes with HTPs is possible should be regulated because they act as important factors affecting changes in smoking behaviors.

## Figures and Tables

**Table 1:** Participants’ intention to quit cigarette smoking within one month in terms of tobacco product use and participants’ characteristics.

Category	Subgroup	Total		Intention to Quit Cigarette Smoking Within One Month	
		N	Weighted % ^2^	N	Weighted % ^3^
**Total**		36,397	100.0	1557	4.7
**Tobacco product use**					
Use of tobacco products	Exclusive cigarette smoker	31,711	82.0	1296	4.5
	Dual smoker with HTPs	2369	9.0	101	4.3
	Dual smoker with e-cigarettes	1488	5.7	104	7.5
	Triple smoker	829	3.3	56	6.4
Cigarette-smoking intensity	Occasional	2783	8.4	418	16.0
	Light daily (<10 CPD)	4903	13.5	271	5.9
	Moderate daily (10~19 CPD)	14,235	42.8	500	3.8
	Heavy daily (≥20 CPD)	14,476	35.3	368	2.7
**Individual characteristics**					
Sex	Male	32,820	91.1	1407	4.7
	Female	3577	8.9	150	5.0
Age (years)	19~29	4076	17.5	227	5.9
	30~39	5389	19.1	275	5.5
	40~49	8395	25.5	316	4.0
	50~59	8582	22.3	350	4.3
	60~69	6118	10.3	247	4.3
	≥70	3,837	5.3	142	3.9
Monthly household income (1000 KRW ^1^)	<2000	8811	16.1	362	4.8
	2000~3000	6063	15.3	265	4.7
	3000~5000	10,833	32.2	460	4.7
	≥5000	10,690	36.5	470	4.6
Education	≤Elementary	4727	6.6	133	2.6
	Jr. high school	4004	7.9	155	3.9
	High school	16,048	46.1	640	4.3
	≥College	11,618	39.4	629	5.7
Residential area	City	20,942	80.2	941	4.8
	Rural	15,455	19.8	616	4.2
**Health-related characteristics**					
Subjective health status	Excellent/good	13,302	38.2	538	4.5
	Fair	17,157	49.1	739	4.8
	Poor/very poor	5938	12.8	280	4.9
Stress level	None at all	7116	15.7	304	5.2
	Very little	19,038	52.9	783	4.4
	Somewhat	8658	26.5	370	4.5
	High	1585	5.0	100	7.3
Monthly alcohol consumption	None	9458	22.2	425	4.8
	Light-moderate	16,137	46.7	723	4.9
	Heavy	10,802	31.1	409	4.3

^1^ KRW: Currency of Korea (1000 KRW is approximately 1 USD). ^2^ Weighted %: column percentage. ^3^ Weighted %: row percentage. Abbreviations: CPD, cigarettes per day; HTPs, heated tobacco products; N, number; CI, confidence interval.

**Table 2:** Association between tobacco product use and intention to quit cigarette smoking within one month.

Category	Subgroup	Intention to Quit Cigarette Smoking Within One Month	
		Crude OR (95% CI)	Adjusted OR (95% CI)
**Tobacco product use**			
Use of tobacco products	Exclusive cigarette smoker	Ref	Ref
	Dual smoker with HTPs	0.97 (0.76–1.23)	0.80 (0.62–1.04)
	Dual smoker with e-cigarettes	1.74 (1.35–2.25)	1.48 (1.13–1.95)
	Triple smoker	1.47 (1.11–1.95)	1.15 (0.85–1.54)
Cigarette-smoking intensity	Occasional	6.89 (5.75–8.24)	7.12 (5.89–8.61)
	Light daily (<10 CPD)	2.25 (1.84–2.74)	2.28 (1.86–2.79)
	Moderate daily (10~19 CPD)	1.42 (1.19–1.68)	1.40 (1.17–1.66)
	Heavy daily (≥20 CPD)	Ref	Ref
**Individual characteristics**			
Sex	Male	0.94 (0.76–1.17)	1.50 (1.20–1.88)
	Female	Ref	Ref
Age (years)	19~29	1.56 (1.19–2.05)	1.44 (1.03–2.02)
	30~39	1.44 (1.10–1.88)	1.33 (0.94–1.88)
	40~49	1.03 (0.79–1.35)	1.08 (0.77–1.50)
	50~59	1.11 (0.85–1.44)	1.26 (0.91–1.73)
	60~69	1.10 (0.83–1.47)	1.26 (0.93–1.71)
	≥70	Ref	Ref
Monthly household income (1000 KRW ^1^)	<2000	Ref	Ref
	2000~3000	0.99 (0.80–1.21)	0.93 (0.75–1.16)
	3000~5000	0.99 (0.82–1.19)	0.93 (0.76–1.14)
	≥5000	0.96 (0.80–1.16)	0.82 (0.67–1.01)
Education	≤Elementary	Ref	Ref
	Jr. high school	1.51 (1.08–2.10)	1.63 (1.16–2.30)
	High school	1.67 (1.28–2.18)	1.75 (1.28–2.41)
	≥College	2.22 (1.70–2.90)	2.24 (1.61–3.11)
Residential area	City	Ref	Ref
	Rural	0.87 (0.76–0.99)	0.98 (0.85–1.13)
**Health-related characteristics**			
Subjective health status	Excellent/good	Ref	Ref
	Fair	1.07 (0.94-1.23)	1.21 (1.04-1.39)
	Poor/very poor	1.10 (0.92-1.32)	1.31 (1.06-1.61)
Stress level	None at all	Ref	Ref
	Very little	0.84 (0.70–0.99)	0.76 (0.63–0.91)
	Somewhat	0.85 (0.70–1.03)	0.76 (0.62–0.94)
	High	1.43 (1.08–1.88)	1.31 (0.98–1.76)
Monthly alcohol consumption	None	Ref	Ref
	Light-moderate	1.02 (0.87–1.20)	0.92 (0.78–1.10)
	Heavy	0.88 (0.74–1.05)	0.91 (0.76–1.10)

^1^ KRW: Currency of Korea (1000 KRW is approximately 1 USD). Abbreviations: CPD, cigarettes per day; HTPs, heated tobacco products; OR, odds ratio; CI, confidence interval.

**Table 3:** Association between tobacco product use and intention to quit cigarette smoking within one month in terms of cigarette-smoking intensity.

Tobacco Product Use	Intention to Quit Cigarette Smoking Within One Month			
	aOR (95% CI)			
	Occasional	Light Daily (<10 CPD)	Moderate Daily (10~19 CPD)	Heavy Daily (≥20 CPD)
Exclusive cigarette smokers (N = 1296)	Ref	Ref	Ref	Ref
Dual smoker with HTPs (N = 101)	0.70 (0.45–1.09)	0.76 (0.41–1.39)	0.75 (0.46–1.20)	1.17 (0.66–2.09)
Dual smoker with e-cigarettes (N = 104)	1.14 (0.70–1.84)	1.81 (1.04–3.14)	1.44 (0.93–2.24)	1.97 (1.14–3.42)
Triple smoker (N = 56)	1.08 (0.61–1.91)	0.71 (0.37–1.35)	1.41 (0.93–2.14)	1.29 (0.61–2.73)

Adjusted for sex, age, monthly household income, education, residential area, subjective health status, stress level, and monthly alcohol consumption. Abbreviations: CPD, cigarettes per day, HTPs, heated tobacco products; aOR, adjusted odds ratio; CI, confidence interval.

## Appendix A

**Table A1:** Number and weighted percentage of intention to quit cigarette smoking according to tobacco product use and cigarette-smoking intensity.

Tobacco Product Use	Intention to Quit Cigarette Smoking Within One Month							
	Occasional (N = 418)		Light Daily (<10 CPD) (N = 271)		Moderate Daily (10~19 CPD) (N = 500)		Heavy Daily (≥20 CPD) (N = 368)	
	n	Weighted%	n	Weighted%	n	Weighted%	n	Weighted%
Exclusive cigarette smokers (N = 1296)	337	15.8	229	5.7	419	3.7	311	2.5
Dual smoker with HTPs (N = 101)	30	13.7	16	5.0	33	2.9	22	3.0
Dual smoker with e-cigarettes (N = 104)	31	19.3	18	10.2	30	5.8	25	4.7
Triple smoker (N = 56)	20	20.5	8	4.5	18	5.5	10	3.3

Abbreviations: CPD, cigarettes per day; HTPs, heated tobacco products; N(n), number.
